# Prevalence of Metabolic Syndrome among Iranian Population: A Systematic Review and Meta-analysis

**Published:** 2017-04

**Authors:** Sahar DALVAND, Seyed Hassan NIKSIMA, Reza MESHKANI, Reza GHANEI GHESHLAGH, Sahar SADEGH-NEJADI, Wesam KOOTI, Naser PARIZAD, Hossein ZAHEDNEZHAD, Reza AFRISHAM

**Affiliations:** 1. Social Determinants of Health Research Center, Kurdistan University of Medical Sciences, Sanandaj, Iran; 2. Dept. of Epidemiology and Biostatistics, School of Public Health, Tehran University of Medical Sciences, Tehran, Iran; 3. Dept. of Clinical Biochemistry, School of Medicine, Tehran University of Medical Sciences, Tehran, Iran; 4. Dept. of Nursing, Faculty of Nursing and Midwifery, Kurdistan University of Medical Sciences, Sanandaj, Iran; 5. Research Committee, Kurdistan University of Medical Sciences, Sanandaj, Iran; 6. Dept. of Medical-Surgical Nursing, School of Nursing and Midwifery, Tabriz University of Medical Sciences, Tabriz, Iran; 7. Dept. of Nursing, University of Social Welfare and Rehabilitation Sciences, Tehran, Iran

**Keywords:** Metabolic syndrome, Prevalence, Meta-analysis, Systematic review, Iran

## Abstract

**Background:**

Metabolic syndrome (MetS) is the major risk factor for development of type 2 diabetes and cardiovascular diseases in different populations. The aim of this study was to evaluate the prevalence of MetS among Iranian population.

**Methods:**

Thirty-four cross-sectional studies were analyzed with a sample of 83227 people. National and international English electronic databases (PubMed, Google scholar, Web of Science, Science Direct, and Scopus) and Persian language databases (SID, Medlib, Iran medex, Magiran, Medlib, and IranDoc) were used to search the articles published on MetS in Iranian population from Jan 2005 to May 2016. The MetS diagnosis was performed according to the ATP-III, NCEP/ATP-III, IDF and WHO criteria.

**Results:**

The overall weighted prevalence of MetS was 31% (95% CI: 28–35). According to ATP III criteria, total and gender-stratified prevalence of MetS in women and men were 29% (95% CI: 22–36), 37% (95% CI: 26–48) and 29% (95% CI: 23–36), respectively. Total prevalence of MetS based on NCEP/ATP III criteria was 29% (95% CI: 24–35) that the prevalence was 24% (95% CI: 18–30) and 35% (95% CI: 25–44) in men and women, respectively. According to the IDF and WHO criteria, total prevalence of MetS were 38% (95% CI: 32–43) and 30% (95% CI: 7–53), respectively.

**Conclusion:**

The findings demonstrate an emerging high prevalence of MetS in total and in particular among Iranian women population. Therefore, to minimize the risk of cardiovascular events in Iranian population, screening and early detection of risk factors for MetS are required.

## Introduction

Metabolic syndrome (MetS) is a collection of different cardiovascular risk factors such as dyslipidemia, hypertension, abdominal obesity and diabetes (1). The concept of MetS has existed for at least 80 yr (2). MetS was first described as X syndrome (3). MetS is linked with several cardiovascular events (4). The prevalence of MetS is increasing globally (5). In Western countries, 23% of the population suffers from this syndrome (6). According to the International Diabetes Federation criteria, one in four adults in the world has MetS. The risk of death, stroke and heart attacks is two to three times more in subjects with MetS compared to individuals without this syndrome (7). More importantly, the prevalence of this disorder is increasing in children and young adults worldwide (8).

MetS have five essential components, including obesity/abdominal obesity, high blood pressure, low high-density lipoprotein cholesterol (HDLC), elevated triglycerides and hyperglycemia. Several definitions have been proposed for MetS and the most commonly used are the Adult Treatment Panel III (ATP-III) and adapted ATP-III criteria (NCEP/ATP-III-A) (9, 10) by the National Cholesterol Education Program (NCEP), the International Diabetes Federation (IDF) (11) and the World Health Organization (WHO) criteria (12).

MetS enhances the risk for various diseases such as diabetes, cardiovascular disease, fatty liver, asthma, ovarian cysts (13) and some cancers (14). To reduce the risk for the mentioned diseases, studying the prevalence of this syndrome seems to be essential in different populations. Accordingly, several studies have investigated the prevalence of MetS in various provinces of Iran (15–19).

In this study, we performed a systematic review and meta-analysis of available cohort studies to determine the prevalence of MetS in Iranian population according to different MetS criteria.

## Methods

### Search strategy

We searched International databases (PubMed, Google scholar, Web of Science, Science Direct and Scopus) and Persian national databases [SID (Scientific Information Database), Medlib (Iranian Medical Library), Iran Medex (articles published in Iran Biomedical Journals), Magiran and IranDoc] for published articles concerning the MetS in Iranian population. The current research was performed using medical subject headings (MeSH) terms and the combination of keywords including: “metabolic syndrome”, “dysmetabolic syndrome”, ” insulin resistance syndrome” with the words “prevalence” and “Iran”. The Persian equivalent of these terms and all possible combinations in the Persian national databases were searched. Articles with English abstract were also used.

### Study selection

Initially, a list of the titles and abstract of all papers contained in databases was prepared to evaluate the relevant titles. Comply with the inclusion criteria; the cross-sectional studies that estimate the prevalence of MetS among Iranian population from Jan 2005 to May 2016 were included in the current research. To assess the eligibility and inclusion criteria, articles were evaluated by two independent reviewers. In the articles, MetS diagnosis was performed according to ATP-III [9], NCEP / ATP-III [10], IDF [11] and WHO [12] criteria. Table 1 shows common definitions of MetS used in the present study.

**Table 1:** Common definitions of metabolic syndrome

	**ATP III Definition (3 of 5 Required)**	**NCEP / ATP III Definition (3 of 5 Required)**	**IDF Consensus Definition (Waist Plus 2 Required)**	**WHO**
Waist circumference	Men > 102 cm (>40 in)	Men > 102 cm (>40 in)	Male ≥ 94 cm (>37 in)	BMI >30 kg/m^2^, or WHR >0.9 in male and>0.85 in female
	Women > 88 cm (>35 in)	Women > 88 cm (>35 in)	Female ≥ 80 cm (>31.5 in)	Plus two or more of the following:
Blood pressure Systolic / diastolic	≥130 / ≥85 mmHg	≥130 / ≥85 mmHg	≥130/≥85 mmHg	≥140 / ≥90 mmHg
HDL cholesterol	Men < 40 mg/dl (1.03 mmol/l)	Men < 40 mg/dl (1.03 mmol/l)	Men < 40 mg/dl (1.03 mmol/l)	Men < 35 mg/dl (0.9 mmol/L)
	Women < 50 mg/dl (1.29 mmol/l)	Women < 50 mg/dl (1.29 mmol/l)	Women < 50 mg/dl (1.29 mmol/l)	Women < 39 mg/dl (1 mmol/L)
Triglycerides	≥150 mg/dl (1.7 mmol/l)	≥150 mg/dl (1.7 mmol/l)	≥150 mg/dl (1.7 mmol/l)	≥150 mg/dl (1.7 mmol/l)
Fasting glucose	≥110 mg/dl (6.1 mmol/l)	≥100 mg/dl (5.6 mmol/l)	≥100 mg/dl (5.6 mmol/l)	Diabetes or impaired glucose tolerance (2-h post load plasma glucose ≥7.8 mmol/L)

BMI= Body Mass Index

WHR= Waist to Hip Ratio

Exclusion criteria in this study were: 1) studies with no original research (reviews, editorials, non-research letters); 2) case reports and case series; 3) studies with non-random sampling method; 4) studies including subjects with hemochromatosis, chronic liver disease, and liver cirrhosis; 5) studies including pregnant women; 6) insufficient data; 7) non-standardized diagnosis; and 8) studies published in Persian without an English abstract.

Overall, 34 related articles were reviewed. Then, the desired data were accurately obtained using a data extraction form on the basis of title, year of publication, type of study, region (province), sample size, the definition used for MetS, gender, and four different criteria for MetS definition. The present study was performed based on PRISMA guideline (Preferred reporting items for systematic reviews and meta-analysis) (20).

### Meta-analysis

Point estimates and their 95% confidence intervals (CI) of the prevalence of MetS was calculated using random-effects model (DerSimonian and Laird’s) and presented in a Forest plot to visualize the heterogeneity among studies. The variance of MetS prevalence in each study was calculated with respect to binomial distribution formula. To study the potential publication bias, assess small study effects, Egger regression test, and funnel plot were used. The Cochran *Q* test was used to investigate the heterogeneity among studies (*P*>0.1 was significant).

The I^2^ index was used to assess the percentage of variation across the studies due to heterogeneity rather than the chance. A value of 0% indicates no observed heterogeneity, while 100% indicates a significant heterogeneity. (Values of 25%, 50%, and 75% were considered representing low, medium and high heterogeneity, respectively). In this study, I^2^ values above 75% were used to show a significant heterogeneity (21, 22). Furthermore, we tested the heterogeneity among subgroups using meta-regression. In addition, meta-regression test was applied to evaluate the relationship between the prevalence of MetS, the year of publication, and the study sample size. Furthermore, we stratified included studies by diagnostic criteria (ATP III, NCEP/ATP III, IDF, WHO) and overall prevalence was estimated according to four definitions. We tested the heterogeneity among subgroups using meta-regression analysis. For the purpose of meta-analysis, included studies were assumed random samples from studies population. The meta-analysis was performed with STATA software ver. 12.0.

## Results

Based on inclusion criteria, 34 cross-sectional studies out of 921 studies had eligibility to be included in current systematic review and meta-analysis and analyzed with a sample of 83227 subjects. Studies including 80 (23) and 12514 subjects (24) were the smallest and largest sample size included in the analyses. The articles included in the meta-analysis were different according to each MetS criteria.

The results of this study indicated the possibility of statistically significant bias using Egger regression test (*P*=0.023). Heterogeneity of the samples was examined by Cochran *Q* test and I^2^ index. The heterogeneity of the samples was significant with *Q*=7987.63 (*P*<0.001) and I^2^ index was 99.4% (*P*<0.001). According to diagnostic criteria total prevalence of MetS was 29% (95% CI: 22–36) and 29% (95% CI: 24–34) based on ATP III criteria and NCEP/ATP III criteria, respectively. In addition, based on IDF and WHO criteria, total prevalence of MetS were 38% (95% CI: 32–43) and 30% (95% CI: 7–53), respectively. In this Meta-analysis, common definitions of metabolic syndrome are presented in Table 1. Table 2 shows the results of the prevalence of MetS according to various diagnostic criteria.

**Table 2:** The prevalence of metabolic syndrome in studies based on various diagnostic criteria

**First author (Reference)**	**Year**	**City (Geographical region)**	**Age**	**Gender**	**Diagnostic criteria**	**Sample size (n)**	**Prevalence (%)**	**Confidence interval 95%**
Jalali R (53)	2009	Kovar Fars (2)	19<	Female/male	ATP III	1402	25.6	23–28
					NCEP/ATPIII		29	27–31
					IDF		30.5	28–33
Sadrbafoghi SM (54)	2006	Yazd (5)	20–74	Female/male	ATP III	1110	32.1	29–35
Gherghereh-chi R (55)	2010	Tehran (1)	4–18	Child/teenager	ATP III	235	31.9	26–38
Shahbazian H (17)	2013	Ahvaz (4)	20–70	Female/male	ATP III	912	22.8	20–26
Javadi H (56)	2014	Qazvin (1)	24<	Female/male	ATP III	996	33	30–36
Marjani A (57)	2011	Gorgan (1)	Uncertain	Female/male	ATP III	200	51.5	54–58
Sharifi F (16)	2009	Zanjan (3)	20<	Female/male	ATP III	2941	23.7	22–25
Jouyandeh Z (58)	2013	Tehran (1)	Uncertain	Postmenopausal women	ATP III	118	30.1	22–38
Hadaegh F (59)	2009	Tehran (1)	65<	Female/male	ATP III	720	50.8	47–54
					IDF		41.9	38–46
					WHO		41.8	38–45
Maharlouei N (60)	2013	Shiraz (2)	40<	Female(Pre-menopause)	ATP III	490	30	26–34
					IDF		32.2	28–36
				Female(Post-menopause)	ATP III		51.2	46–56
					IDF	434	53.2	49–58
Salem Z (15)	2007	Rafsanjan (5)	11–18	Female	ATP III	1221	3.9	3–5
Mardani M (61)	2015	Khorramabad (4)	19–27	Female/male	ATP III	214	1.9	0.1–7.3
Jalalzadeh M (23)	2011	Zanjan (3)	16<	Female/male	ATP III	80	28.7	19–39
Sarrafzadegan N (62)	2011	Isfahan (2)	19<	Female/male	ATP III	9570	22.5	22–23
Kaykhaei M (63)	2012	Zahedan (5)	19<	Female/male	NCEP/ATPIII	1802	21	19–23
					IDF		24.8	23–27
Fakhrzadeh H (64)	2006	Tehran (1)	25–64	Female/male	NCEP/ATPIII	1480	27.5	25–30
Foroozanfar Z (65)	2015	Kerman (5)	Uncertain	Female/male	NCEP/ATPIII	950	73.4	71–76
					IDF		64.9	62–68
Keykha M (66)	2013	Isfahan (2)	30–60	Female/male	NCEP/ATPIII	3228	35.8	34–37
Rashidi H (49)	2014	Ahvaz (4)	10–19	Female/male	NCEP/ATPIII	2246	9	8–10
Marjani A (67)	2012	Gorgan (1)	20<	Female	NCEP/ATPIII	160	20.6	14–27
Esmailnasab N (68)	2012	Kurdistan (3)	25–64	Female/male	NCEP/ATPIII	1194	29.1	27–32
		National survey						
Zabetian A (42)	2007	Tehran (1)	20<	Female/male	NCEP/ATPIII	10368	33.2	32–34
					IDF		32.1	31–33
					WHO		18.4	18–19
Marjani A (69)	2012	Gorgan (1)	>45	post-menopause	NCEP/ATPIII	100	31	22–40
Ostovaneh M (70)	2014	Zahedan (5)	16<	Female/male	NCEP/ATPIII	2243	12	11–13
					IDF		11.8	10–13
		Amol (1)	16<	Female/male	NCEP/ATPIII	5826	27.8	27–29
					IDF		26.9	26–28
Gharipour M (24)	2011	Isfahan (2)	19<	Female/male	NCEP/ATPIII	12514	23.2	22–24
Ghorbani R (71)	2012	Semnan (1)	30–70	Female/male	NCEP/ATPIII	3799	28.5	27–30
					IDF		35.8	34–37
Hajian-Tilaki K (37)	2014	Babol (1)	20–70	Female/male	NCEP/ATPIII	1000	42.3	39–45
Tabatabaei AH (72)	2015	Shiraz (2)	20<	Female/male	NCEP/ATPIII	377	26.8	22–31
Saberi H (19)	2009	Kashan (2)	30<	Man driver	NCEP/ATPIII	429	35.9	31–40
Delavar MA (50)	2009	Babol (1)	30–50	Female	NCEP/ATPIII	916	31	28–34
Mahjoub S (73)	2012	Babol (1)	20<	Female/male	NCEP/ATPIII	933	23.7	21–26
Moini A (74)	2012	Tehran (1)	15–40	Female/male	NCEP/ATPIII	282	23	18–28
Ebrahimi-Mameghani M (75)	2011	Tabriz (3)	Uncertain	Male firefighter	IDF	76	56.6	45–68
				Male employees		73	60.3	49–72
Mohebbi I (76)	2012	Zanjan (3)	20–67	Man driver	IDF	12138	32.4	32–33

The highest and lowest prevalence of MetS were found in Kerman 73.4% (95% CI: 71–76) and Khorramabad 1.9% (95% CI: 0.1–7.3), respectively. Table 3 shows the prevalence of MetS according to gender and age (less and more than 19 yr of age) groups. On basis ATP III criteria, women and men had a prevalence of 37% (95% CI: 26–48) and 29% (95% CI: 23–36), respectively. Based on the NCEP/ATP III criteria, the prevalence of MetS were 24% (95% CI: 18–30) and 35% (95% CI: 25–44) men and women groups, respectively. Also, the prevalence of MetS at ages less than 19 yr and the age group above 19 yr were 18% (CI 95%: 12% – 25%) and 31% (CI 95%: 28% – 34%), respectively.

**Table 3:** The prevalence of metabolic syndrome according to gender and age in the meta-analysis

	**Diagnostic criteria**	**Number of studies**	**Prevalence %**	**Confidence interval**	**Homogeneity (I**^**2**^**)**	***P*-value**
women		11	37	26–48	99.5	
men		8	29	23–36	97.1	
^*‡*^*PP gender*		19	34	27–41	99.2	
age < 19	ATP III	2	17	10–45	98.8	*P* <0.001
age ≥ 19		9	29	22–36	99.1	
*PP age*		11	27	20–35	99.4	
women		12	35	25–44	99.5	
men		13	24	18–30	98.8	
*PP gender*	NCEP/	25	29	24–35	99.4	*P* <0.001
age < 19	ATP III	4	17	8–27	99.5	
age ≥ 19		12	29	26–32	97.7	
*PP age*		16	26	22–31	99.2	
women		6	42	30–54	99.5	
men		7	32	25–38	98.9	
*PP gender*	IDF	13	37	31–43	99.3	*P* <0.001
age < 19		3	21	11–31	99.3	
age ≥ 19		6	36	33–39	99.4	
*PP age*		9	32	27–37	99.1	
women	WHO	1	47	41–52	--	
men		1	38	34–43	--	--

‡ Pooled prevalence

Table 4 shows subgroups analysis of the prevalence of MetS in different regions across the diagnostic criteria. Based on geography classification, Iran is divided into 5 regions. Region 1 includes the provinces of Tehran, Qazvin, Golestan, Mazandaran, Semnan, Alborz, and Qom. Region 2 contains Isfahan, Fars, Bushehr, Chaharmahal and Bakhtiari, Hormozgan and Kohkiluyeh and Boyerahmad provinces. Region 3 covers East Azerbaijan, West Azerbaijan, Ardabil, Zanjan, Gilan and Kurdistan provinces. Region 4 includes Kermanshah, Ilam, Lorestan, Hamadan, Markazi and Khuzestan provinces, and region 5 contains Razavi Khorasan, South Khorasan, North Khorasan, Kerman, Yazd and Sistan-Baluchestan provinces (25). Total prevalence of MetS in the region 1, 2, 3, 4 and 5 were 31% (CI 28–35%), 33% (CI 29–36%), 33% (CI 21–45%), 11% (CI 2–20%) and 38% (CI 20–56%), respectively (Table 4). Based on meta–regression test, the prevalence of MetS is reduced by increasing the sample size. Due to the positive slope of the meta-regression line and *P-*value=0.175, the prevalence of MetS has no significant relation with the sample size in Iran. Furthermore the prevalence of MetS is increased with increasing the year of the study, but the difference was not statistically significant (*P*=0.604).

**Table 4:** Subgroup analysis for comparison of prevalence in different region across diagnostic criteria

**Region**	**Method**	**No. of studies**	**Prevalence (95% CI)**	**I^2^ %**	**Heterogeneity test**		**Egger test**	
					***Q***	***P***	***t***	***P***
	ATP III	5	40 (30–49)	94.9	78.55	*P* <0.001	0.12	0.911
	NCEP	10	29 (26–32)	94.7	169.49	*P* <0.001	0.74	0.480
1	IDF	5	30 (21–38)	99.5	806.62	*P* <0.001	0.15	0.891
	WHO	2	30 (7–53)	99.4	155.40	*P* <0.001	---	---
Total	---	22	31 (28–35)	98.9	1922	*P* <0.001	1.54	0.138
	ATP III	4	32 (23–41)	98	151.1	*P* <0.001	2.30	0.148
2	NCEP	5	30 (24–37)	98.1	214.11	*P* <0.001	1.42	0.250
	IDF	3	38 (26–51)	97.2	72.72	*P* <0.001	1.17	0.449
Total	---	12	33 (29–36)	98.0	544.04	*P* <0.001	3.71	0.004
	ATP III	3	18 (22–35)	99.5	438.15	*P* <0.001	0.47	0.721
3	NCEP	1	29 (27–32)	---	---	---	---	---
	IDF	3	49 (28–70)	95.2	41.40	*P* <0.001	14.82	0.043
Total	---	7	33 (21–45)	99.7	1778.21	*P* <0.001	0.27	0.798
	ATP III	2	12 (8–33)	99.4	155.95	*P* <0.001	--	--
4	NCEP	1	9 (7–10)	--	--	--	--	--
	IDF	0	--	--	--	--	--	--
Total	---	3	11 (2–20)	98.7	156.32	*P* <0.001	0.50	0.702
	ATP III	1	32 (29–35)	--	--	--	--	--
5	NCEP	3	35 (5–66)	99.9	1497.04	*P* <0.001	3.23	0.191
	IDF	2	45 (6–84)	99.8	468.42	*P* <0.001	--	--
Total	---	6	38 (20–56)	99.8	2181.50	*P* <0.001	3.94	0.017
	ATP III	15	29 (22–36)	99.3	2009.74	*P* <0.001	1.96	0.071
Total	NCEP	20	29 (24–34)	99.4	2940.51	*P* <0.001	1.08	0.296
	IDF	13	38 (32–43)	99.2	1570.91	*P* <0.001	1.22	0.250
	WHO	2	30 (7–53)	99.4	155.40	*P* <0.001	--	--

On the basis classification of geography, Iran is divided into 5 regions. Region 1 includes the provinces of Tehran, Qazvin, Golestan, Mazandaran, Semnan, Alborz, and Qom. Region 2 includes Isfahan, Fars, Bushehr, Chaharmahal and Bakhtiari, Hormozgan and Kohkiluyeh and Boyerahmad provinces. Region 3 contains the provinces of East Azerbaijan, West Azerbaijan, Ardebil, Zanjan, Gilan, and Kurdistan. Region 4 covers Kermanshah, Ilam, Lorestan, Hamedan, Markazi and Khuzestan, and, region 5 includes Razavi Khorasan, South Khorasan, North Khorasan, Kerman, Yazd and Sistan-Baluchistan provinces

## Discussion

Metabolic syndrome or insulin resistance syndrome is a collection of risk factors for heart diseases including abnormal blood lipids (dyslipidemia), glucose intolerance, central obesity, and hypertension (26). Genetic, metabolic, stress and environmental factors such as the diet play an important role in the development of the syndrome. Since MetS significantly increase the risk of various diseases such as cardiovascular disease, diabetes, ovarian cysts, fatty liver, asthma (13) and some cancers (14), investigating the prevalence of this syndrome seems to be essential in different populations.

According to results of our study, the overall weighted prevalence of this syndrome, excluding the diagnostic criteria was 31% (CI 95%: 28–35). The prevalence of MetS in few studies is between 10% and 30%; however, many other studies have estimated a prevalence of more than 30%. In line with this finding, a high incidence of this syndrome has been observed in the neighboring countries of Iran such as Pakistan and Turkey (27, 28). Based on the results of this study in Iranian population, the prevalence of the syndrome is higher than many countries, including France (25% in men and 15.3% women) (29), America (22.9%) (30), Portugal (27.6%) (31), Spain (26.6%) (32) and Italy (22% in men and 18% in women) (33). However, the prevalence of the MetS in North African countries (30%) (34), Turkey (36.6%) (35) and Colombia (34.8%) (36) is similar to Iran. The difference in the prevalence of MetS across the countries can be primarily attributed to the differences in lifestyles and culture (37).

Based on the diagnostic criteria, the highest prevalence of MetS was based on the IDF with a value of 38% (CI 95%: 7–53%) and the lowest prevalence was obtained from the ATPIII 29% (CI 95%: 7–53%) and NCEP/ATPIII 29% (CI 95%: 7–53%). According to the WHO criteria, the prevalence of this syndrome was calculated 30% (CI 95%: 7–53%). The results of several studies have indicated a higher prevalence of the syndrome based on IDF criterion compared to the other criteria (38, 39). For instant, a high prevalence of MetS according to IDF criteria was reported in a recent study in Germany. In this study, the prevalence of this syndrome according to criteria of IDF was 51% (40). In a study conducted in American adults, the prevalence of the MetS according to IDF criteria was 39% (38.1% in women and 39.9% men) (41). The reason for high prevalence of the MetS using the IDF criteria can be due to a lower cutoff point of waist circumference (39, 42, 43) and tighter criteria for fasting glycaemia (44). However, a lower prevalence of MetS according to the IDF criteria was also reported (45). The highest and lowest prevalence of the MetS were defined by WHO and IDF criteria, respectively (46). The discrepancy between the results of different studies can be contributed to difference in abdominal obesity and waist circumference in different populations.

The prevalence of the syndrome in Iran differs from the neighbors from 20%–37.2% and 32%–47% in men and women, respectively (45). Total prevalence of MetS in men and women were 28% and 38%, respectively. In all diagnostic criteria, the prevalence of the MetS was significantly higher in women than men. Similar to our findings, reports from Unite state of America (Native and Mexican American), Turkey, Oman and India demonstrate a higher rate of MetS prevalence in women compared to men (47). A higher rate of the MetS in women can be attributed to increasing of abdominal obesity in this group. Increased abdominal obesity is due to a lower level of physical activity, higher order of birth and menopause status (48).

The prevalence of MetS is highly depended on age has been observed in many studies (48–50). The results of this study also show that the prevalence of MetS in aged 4–90 yr in both sexes and in different regions varies from 1% to 74%. Most of gender difference in the prevalence of the MetS is observed in higher age groups. In this meta-analysis, the prevalence rate of MetS using a random effects model, at ages less and more than 19 yr were 18% (CI 95%: 12% – 25%) and 31% (CI 95%: 28% – 34%), respectively. The results show an upward trend according to age in both sexes and the prevalence was enhanced in all diagnostic criteria. In agreement with our findings, the prevalence of the syndrome was less than 10% for men and women in the age group below 30 yr, but the prevalence has increased in age group 60–69 yr more than 38% and 67% in men and women, respectively (51). In a French population, the prevalence of this syndrome was less than 5.6% in the age group 30–39 yr, but it was increased to over 17.5% in the age group over 60 yr. In addition, in a study in America, the prevalence of MetS were 7%, 44% and 42% in the age group under 30 yr, 69–60 yr and older than 70 yr, respectively (52). In contrast to our results, the prevalence of MetS decreased from 25.5% to 22.9%. Furthermore, in this study a significant increase was calculated in the prevalence of the MetS with increasing age in women (*P*=0.005) but not in men (*P*=0.54) (30).

Based on the geographical region, the highest and lowest prevalence of the MetS were observed in the regions 5 (38%) and 4 (11%), respectively. A relatively large change in the prevalence of the syndrome has been observed in region 4 in comparison to the other regions. More specifically, the residents of Kerman, Tehran, Shiraz, Tabriz and Zahedan, Iran demonstrated a higher prevalence of the MetS based on all diagnostic criteria. The discrepancies among the regions may be associated with different factors including lifestyle, socioeconomic status, nutritional status and education of the residents of these areas. It is of note that the modern lifestyles such as consuming fast foods of high-calorie play an important role in development of the MetS and obesity.

## Advantages and Limitations

The high heterogeneity observed among studies could be regarded as a limitation of the present study. Probably the small sample size of a few of the studies included in the current review could be regarded as the reason of the heterogeneity. Second, due to limited access to some Persian articles, some studies might have been missed. Nevertheless, the main strengths of this study were that most of the included studies had large sample sizes. Two investigators independently extracted data and reviewed the articles to obtain data accurately.

## Conclusion

The prevalence of the MetS in Iranian population is higher than the western counterparts. There is an emerging high prevalence of MetS in Iranian women population. In addition, the prevalence of MetS increases with increasing the age in this population. Therefore, to reduce the risk of cardiovascular events in Iranian population, screening and early detection of risk factors for MetS are suggested.

## Ethical considerations

Ethical issues (Including informed consent, plagiarism, misconduct, data fabrication and/or falsification, double publication and/or submission, redundancy, etc.) have been completely observed by all of the authors.
